# Myostatin regulates the production of fibroblast growth factor 23 (FGF23) in UMR106 osteoblast–like cells

**DOI:** 10.1007/s00424-021-02561-y

**Published:** 2021-04-25

**Authors:** Franz Ewendt, Martina Feger, Michael Föller

**Affiliations:** 1 Institute of Agricultural and Nutritional Sciences, Martin Luther University Halle-Wittenberg, 06120 Halle (Saale), Germany; 2 Department of Physiology, University of Hohenheim, Garbenstraße 30, 70599 Stuttgart, Germany

**Keywords:** TGF-β, p38MAPK, Ca^2+^, Vitamin D, Phosphate

## Abstract

Myostatin is a signaling molecule produced by skeletal muscle cells (myokine) that inhibits muscle hypertrophy and has further paracrine and endocrine effects in other organs including bone. Myostatin binds to activin receptor type 2B which forms a complex with transforming growth factor-β type I receptor (TGF-βRI) and induces intracellular p38MAPK and NFκB signaling. Fibroblast growth factor 23 (FGF23) is a paracrine and endocrine mediator produced by bone cells and regulates phosphate and vitamin D metabolism in the kidney. P38MAPK and NFκB-dependent store-operated Ca^2+^ entry (SOCE) are positive regulators of FGF23 production. Here, we explored whether myostatin influences the synthesis of FGF23. *Fgf23* gene expression was determined by qRT-PCR and FGF23 protein by ELISA in UMR106 osteoblast–like cells. UMR106 cells expressed activin receptor type 2A and B. Myostatin upregulated *Fgf23* gene expression and protein production. The myostatin effect on *Fgf23* was significantly attenuated by TGF-βRI inhibitor SB431542, p38MAPK inhibitor SB202190, and NFκB inhibitor withaferin A. Moreover, SOCE inhibitor 2-APB blunted the myostatin effect on *Fgf23*. Taken together, myostatin is a stimulator of *Fgf23* expression in UMR106 cells, an effect at least partially mediated by downstream TGF-βRI/p38MAPK signaling as well as NFκB-dependent SOCE.

## Introduction

Myostatin is part of a group of signaling molecules produced by skeletal muscle cells (myocytes) that are known under the name “myokines” in analogy to “cytokines” [25]. It was discovered in 1997 as a member of the TGF-β superfamily and first named “growth differentiation factor 8 (GDF-8)” [33]. Myostatin counteracts muscle hypertrophy as mice deficient in myostatin exhibit 2–3 times more muscle mass than wild-type animals [33]. Also the Belgian Blue, cattle with a loss of function mutation in the gene encoding myostatin, is characterized by enormous muscle mass [16, 25]. Finally, mutations in the human gene encoding myostatin, which result in muscle hypertrophy, are similarly described [25, 41].

In addition to myocytes, myostatin also affects bone metabolism including bone formation and osteoclastogenesis [25]. In osteocytes, myostatin upregulates sclerostin and dickkopf-related protein 1 (Dkk1) [25]. Thus, the myokine myostatin plays a role as regulatory molecule in the cross-talk between muscle and bone [25, 29].

Activin receptor type 2A and B (ACVR2A/B) are the membrane receptors for myostatin. Upon binding of myostatin, transcription factor activity of SMAD2/3 and forkhead box O (FOXO) is induced, ultimately resulting in the degradation of skeletal muscle proteins [17]. Further downstream effectors of myostatin also include p38MAPK [35].

Fibroblast growth factor 23 (FGF23) can be considered as an osteokine, a hormone mainly synthesized in the bone [25, 43]. Having classical endocrine effects, its main target organ is the kidney where it binds to a membrane receptor, which is dependent on transmembrane protein αKlotho [19], and inhibits the formation of calcitriol (1,25(OH)_2_D_3_, biologically active vitamin D_3_) by suppressing 25-hydroxyvitamin d-1α-hydroxylase (encoded by *CYP27B1*), the renal key enzyme for 1,25(OH)_2_D_3_ synthesis. Moreover, FGF23 fosters the internalization of membrane NaP_i_IIa (*SLC34A1*), the major Na^+^-dependent phosphate cotransporter in the proximal tubule of the kidney, thereby increasing the urinary excretion of phosphate [20, 39].

FGF23 may also be produced locally by other cells including hepatocytes [32] or cardiomyocytes thus exerting additional paracrine effects [28]. Thus, it was shown that FGF23 affects cardiac muscle and induces left ventricular hypertrophy [12], which underlines that this bone-derived molecule participates in the cross-talk between bone and muscle [25].

Moreover, the plasma concentration of FGF23 goes up in many acute and chronic diseases, notably renal and cardiovascular disorders [3, 21, 42]. Interestingly, myostatin is also upregulated in an early stage of chronic kidney diseases (CKD) [46]. Whether FGF23 is only a disease biomarker or actively drives pathophysiological processes is not entirely clear, yet [42].

The regulation of the production of FGF23 is subject to current research. Well-established regulators of FGF23 include 1,25(OH)_2_D_3_ [31], dietary phosphate [44], parathyroid hormone [27], pro-inflammatory cytokines and pathways [8, 14], interleukin-6 (IL-6) [7], erythropoietin [5, 18], iron metabolism [6], transforming growth factor (TGF)-β (TGF-β) [13], peroxisome proliferator-activated receptor α [11], and intracellular signaling cascades including AMP-dependent protein kinase (AMPK) [15] and insulin/IGF-1-dependent phosphoinositide 3-kinase/Akt/FOXO signaling [2]. Remarkably, myostatin downstream signaling effector p38MAPK also controls *Fgf23* gene expression [10].

Some human diseases with enhanced myostatin plasma levels including dermatomyositis and CKD are characterized by enhanced FGF23 production [23, 46]. This and the well-established muscle and bone cross-talk [25] prompted us to investigate whether myostatin directly impacts on FGF23 formation and to uncover the underlying mechanism. A direct effect could be of high relevance given that pharmacological manipulation of myostatin in disease may be a future option [25].

## Methods

### Cell culture and treatments

UMR106 rat osteoblast-like cells (CRL-1661; ATCC, Manassas, VA, USA) were cultured under standard conditions [13]. Per se, basal *Fgf23* expression is low in UMR106 cells [40]. Therefore, they first have to be treated for 24 h with 100 nM 1,25(OH)_2_D_3_ (Tocris, Bristol, UK), which strongly enhances *Fgf23* expression [40]. Next, cells were treated for further 24 h with recombinant myostatin protein (5–100 ng/mL, PeproTech, Rocky Hill, NJ, USA) in the presence or absence of TGF-β type I receptor (TGF-βRI) inhibitor SB431542 (10 µM, Sigma-Aldrich, Schnelldorf, Germany), p38MAPK inhibitor SB202190 (10 µM, Tocris), NFκB inhibitor withaferin A (500 nM, Tocris), or SOCE inhibitor 2-APB (150 µM, Sigma).

IDG-SW3 mouse osteocytes (CVCL_0P23; Kerafast, Boston, MA, USA) were plated on rat tail type I collagen–coated 12-well plates (150,000 cells per well) in α-Minimum Essential Medium (α-MEM) supplemented with 10% fetal bovine serum (FBS), 100 U/mL penicillin, 100 μg/mL streptomycin, and 50 U/mL interferon (IFN)-γ (all reagents from Gibco, Life Technologies, Darmstadt, Germany). Cells were grown for 24 h at 33 °C and 5% CO_2_. Next, differentiation was induced by replacing IFN-γ with 50 μg/ml ascorbic acid (Sigma-Aldrich) and 4 mM β-glycerophosphate (AppliChem, Darmstadt, Germany) and further incubation at 37 °C and 5% CO_2_. The medium was changed every 2nd to 3rd day. At day 28, cells were incubated with 100 ng/mL recombinant myostatin or vehicle in duplicate for 8 h.

MC3T3-E1 Subclone 4 mouse pre-osteoblast cells (CRL-2593; ATCC) were cultured in α‐MEM with 2 mM l-glutamine, nucleosides (Gibco, Life Technologies), 10% FBS, 100 U/mL penicillin, and 100 µg/mL streptomycin. They were studied from passages 23 to 27. To this end, cells were seeded on rat tail type I collagen–coated 12-well plates (80,000 cells per well) for 24 h and incubated with 50 μg/mL ascorbic acid, and 4 mM β-glycerophosphate for 6 days. Then, 100 ng/mL myostatin or vehicle only was added in the presence of 1,25(OH)_2_D_3_ (10 nM) 24 h before harvesting the cells.

### Quantitative real-time polymerase chain reaction

Total RNA was isolated with TriFast reagent (Peqlab, Erlangen, Germany), and 1.2 µg was used along with random primers and the GoScript™ Reverse Transcription System (Promega, Mannheim, Germany) for cDNA synthesis (program: 25 °C for 5 min, 42 °C for 1 h, and 70 °C for 15 min). Quantitative real-time polymerase chain reaction (qRT-PCR) using the Rotor-Gene Q cycler (Qiagen, Hilden, Germany) and GoTaq qPCR Master Mix (Promega) was performed. The amplification conditions for analysis of *Fgf23* and *TATA box-binding protein* (*Tbp*) were 95 °C for 3 min, 40 cycles of 95 °C for 10 s, 57 °C for 30 s, 72 °C for 30 s (in UMR106 cells), and 95 °C for 3 min, 40 cycles of 95 °C for 10 s, 58 °C for 30 s (*Fgf23*), and 60 °C for 30 s (*Tbp*), 72 °C for 30 s (in IDG-SW3 and MC3T3-E1 cells). QRT-PCR conditions for analysis of *Acvr2a* and *Acvr2b* expression in UMR106, IDG-SW3, and MC3T3-E1 cells were 95 °C for 3 min, 40 cycles of 95 °C for 10 s, 60 °C for 30 s, and 72 °C for 30 s. The calculated *Fgf23*, *Acvr2a*, and *Acvr2b* mRNA transcript levels in UMR106, IDG-SW3, and MC3T3-E1 cells were normalized to the transcript levels of *Tbp.*

The *Acvr2a* and *Acvr2b* qRT-PCR products of the UMR106 cells were loaded on a 1.5% agarose gel and visualized by Midori Green.

The following primers (5′ → 3′ orientation) were used:

- *Rat Acvr2a:*
- F: CAATATCTCACAGGGACATC,
- R: TTTGGAAGTTTATAGCACCC;
- *Rat Acvr2b:*
- F: AACATCATCACGTGGAAC,
- R: AACATTCTTGCTTTTGAAGTC;
- *Rat Fgf23:*
- F: TAGAGCCTATTCAGACACTTC,
- R: CATCAGGGCACTGTAGATAG;
- *Rat Tbp:*
- F: ACTCCTGCCACACCAGCC,
- R: GGTCAAGTTTACAGCCAAGATTCA;
- *Mouse Acvr2a:*
- F: GGTCTCTTGGAATGAACTTTG,
- R: TTACTTTTGATGTCCCTGTG;
- *Mouse Acvr2b:*
- F: ATTACCTCAAGGGGAACATC,
- R: CATTCTTGCTTTTGAAGTCC;
- *Mouse Fgf23:*
- F: TCGAAGGTTCCTTTGTATGGA,
- R: AGTGATGCTTCTGCGACAAGT;
- *Mouse Tbp:*
- F: CCAGACCCCACAACTCTTCC,
- R: CAGTTGTCCGTGGCTCTCTT.

### Enzyme-linked immunosorbent assay

UMR106 cells were cultured as described and treated with 100 ng/mL myostatin for 24 h. The cell culture supernatant was stored at − 80 °C. C-terminal FGF23 was determined by an ELISA kit (Mouse/Rat FGF-23 (C-Term), Immuntopics, San Clemente, CA, USA) according to the manufacturer’s protocol. Intact FGF23 was determined in the supernatant after its concentration by means of Sartorius Vivaspin 6 Centrifugal Concentrators (Sartorius, Göttingen, Germany), with an ELISA kit (Mouse/Rat FGF-23 (Intact), Immuntopics).

### Statistics

The data are shown as arithmetic means ± SEM, and *n* represents the number of independent experiments. Normal distribution was tested by Shapiro–Wilk normality test. Two groups were compared by unpaired Student’s *t* test (if necessary with Welch’s correction) or with Mann–Whitney-*U*-test for data not normally distributed. More than two groups were tested for significance by one-way ANOVA followed by Bonferroni’s multiple comparisons test (if necessary with Welch’s ANOVA followed by Dunnett’s T3 multiple comparisons test). Differences were considered significant if *p* < 0.05.

## Results

### Expression of the activin type 2 receptors in UMR106 cells

**Fig. 1:**
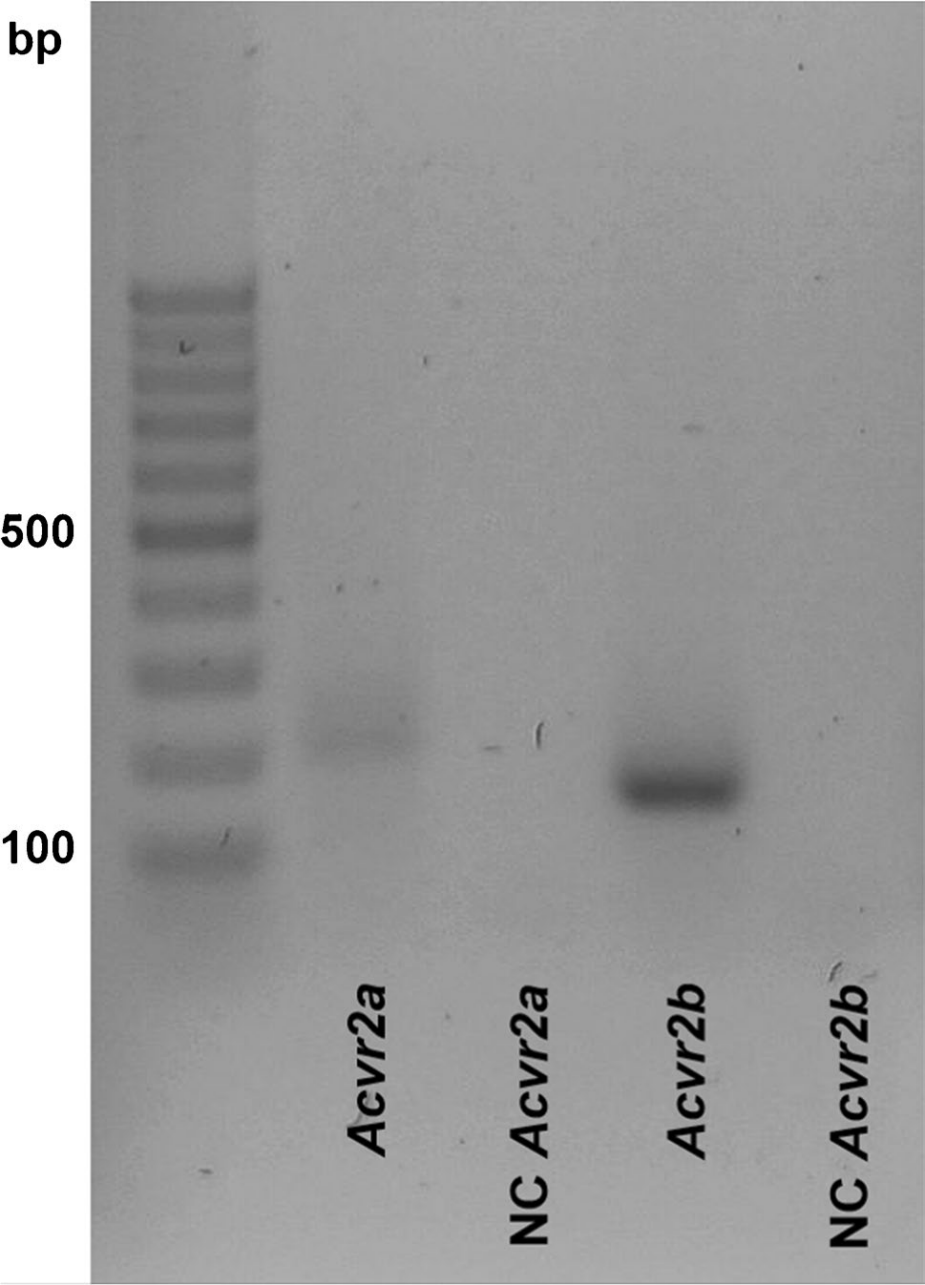
Expression of activin type 2 receptor isoforms in rat UMR106 osteoblast–like cells. Original agarose gel photo showing cDNA specific for activin type 2 receptor A (Acvr2a) and activin type 2 receptor B (Acvr2b) in UMR106 cells. NC, non-template control

The impact of myostatin on *Fgf23* expression was studied in UMR106 osteoblast–like cells. Employing RT-PCR, we first investigated the expression of myostatin receptors activin type 2A and B (*Acvr2a* and *Acvr2b*). As illustrated in Fig. 1, mRNA specific for receptor *Acvr2b* and to a markedly lesser extent for *Acvr2a* could be detected in UMR106 cells. Other cell lines used to study Fgf23 include IDG-SW3 and MC3T3-E1 cells. We used quantitative RT-PCR to compare *Acvr2a* and *Acvr2b* expression in all three cell lines. As a result, expression of *Acvr2a* relative to *Tbp* was 0.0008 ± 0.0000 in UMR106 cells, an expression level significantly (*p* < 0.001) lower than in IDG-SW3 cells (0.6014 ± 0.0615) and MC3T3-E1 cells (0.2768 ± 0.0162; for all *n* = 7). In contrast, expression of *Acvr2b* relative to *Tbp* was 0.5797 ± 0.0465 in UMR106 cells, an expression level significantly (*p* < 0.001) higher than in IDG-SW3 cells (0.0054 ± 0.0009) and MC3T3-E1 cells (0.0170 ± 0.0017; for all *n* = 7).

### Myostatin induces Fgf23 expression in UMR106 cells

**Fig. 2:**
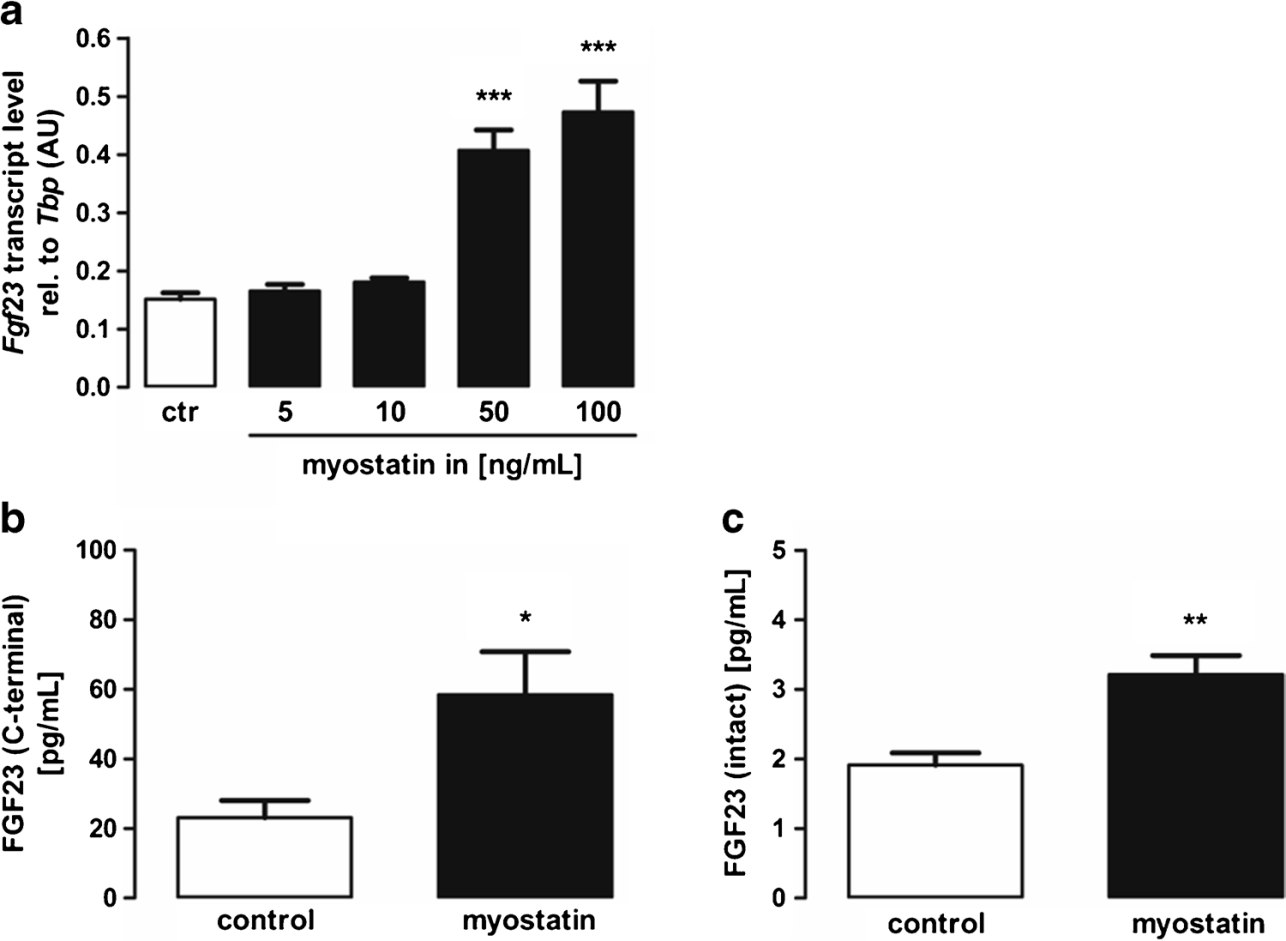
Myostatin induces *Fgf23* expression in UMR106 cells, **a** Arithmetic means ± SEM of relative (rel.) *Fgf23* mRNA abundance or **b** C-terminal or **c** intact FGF23 protein concentration in the cell culture supernatant of UMR106 osteoblast–like cells incubated without (white bars) or with (black bars) myostatin (**a** indicated concentrations, *n* = 7; and **b**, **c** 100 ng/mL, *n* = 10) for 24 h. *p < 0.05, **p < 0.01, ***p < 0.001 indicate significant difference from control. AU, arbitrary units; ctr, control (**a** one-way ANOVA; **b** unpaired Student’s *t* test with Welch’s correction; **c** Mann–Whitney-*U*-test)

Next, we examined whether myostatin influences *Fgf23* expression. To this end, UMR106 cells were incubated without or with different concentrations of myostatin for 24 h and *Fgf23* gene expression was analyzed by quantitative RT-PCR. As shown in Fig. 2a, myostatin significantly increased *Fgf23* gene expression in a dose-dependent manner. Using ELISA, we tested whether the stimulatory effect of myostatin on *Fgf23* gene expression is translated into enhanced FGF23 protein secretion into the cell culture supernatant. Indeed, 24 h treatment with myostatin also upregulated C-terminal FGF23 (Fig. 2b) and intact FGF23 (Fig. 2c) production. In IDG-SW3 cells, an 8 h treatment with 100 ng/mL myostatin resulted in a relative *Fgf23* expression of 0.0006 ± 0.0004 (*n* = 4), a level not significantly different from that in control cells (0.0006 ± 0.0002; *n* = 4). Similarly, a 24 h treatment with 100 ng/mL myostatin led to a relative *Fgf23* expression of 0.0382 ± 0.0025 (*n* = 7) in MC3T3-E1 cells, a level not significantly different from that in control-treated cells (0.0401 ± 0.0042; *n* = 7).

### Transforming growth factor-β type I receptor is necessary for myostatin-induced *Fgf23* expression

**Fig. 3:**
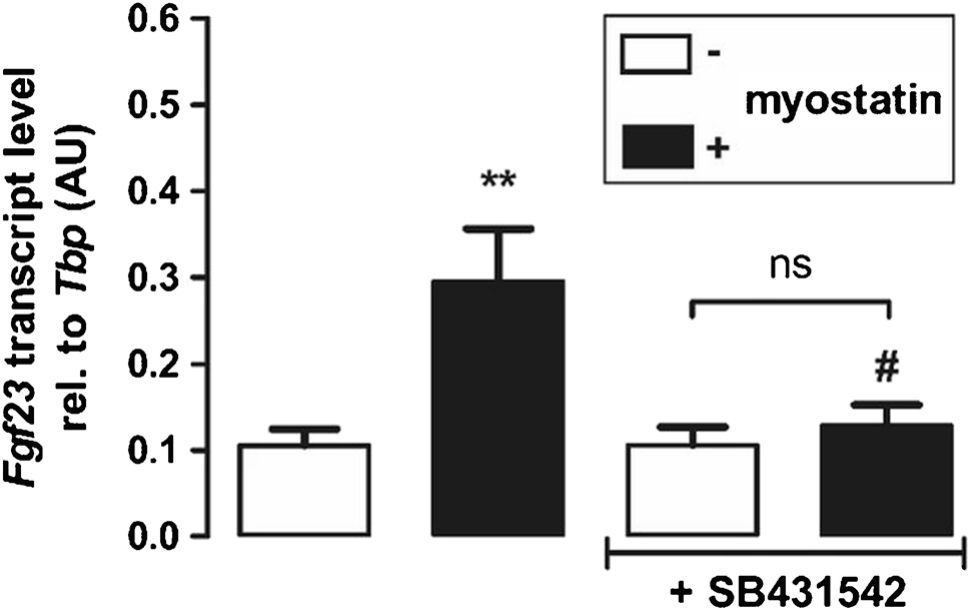
Transforming growth factor-β type I receptor (TGF-βRI) is necessary for myostatin-induced *Fgf23* gene expression. Arithmetic means ± SEM of relative (rel.) *Fgf23* mRNA abundance in UMR106 osteoblast–like cells incubated without (white bars) or with (black bars) myostatin (100 ng/mL, 24 h, *n* = 7) in the presence or absence of TGF-βRI inhibitor SB431542 (10 µM, 24 h). **p < 0.01 indicates significant difference from control. #*p* < 0.05 indicates significant difference from the absence of SB431542 (2nd vs 4th bar). AU, arbitrary units (one-way ANOVA)

The next experiments explored the signaling of the myostatin effect on *Fgf23*. The binding of myostatin to ACVR2B induces complex formation with TGF-βRI [38]. In order to study whether this process is involved, we incubated UMR106 cells with 100 ng/mL myostatin in the presence or absence of TGF-βRI inhibitor SB431542 (10 µM) for 24 h. SB431542 treatment significantly abrogated myostatin-induced *Fgf23* expression in UMR106 cells (Fig. 3).

### The myostatin effect on ***Fgf23*** is at least in part dependent on p38MAPK and NFκB-mediated store-operated Ca^2+^ entry (SOCE)

**Fig. 4:**
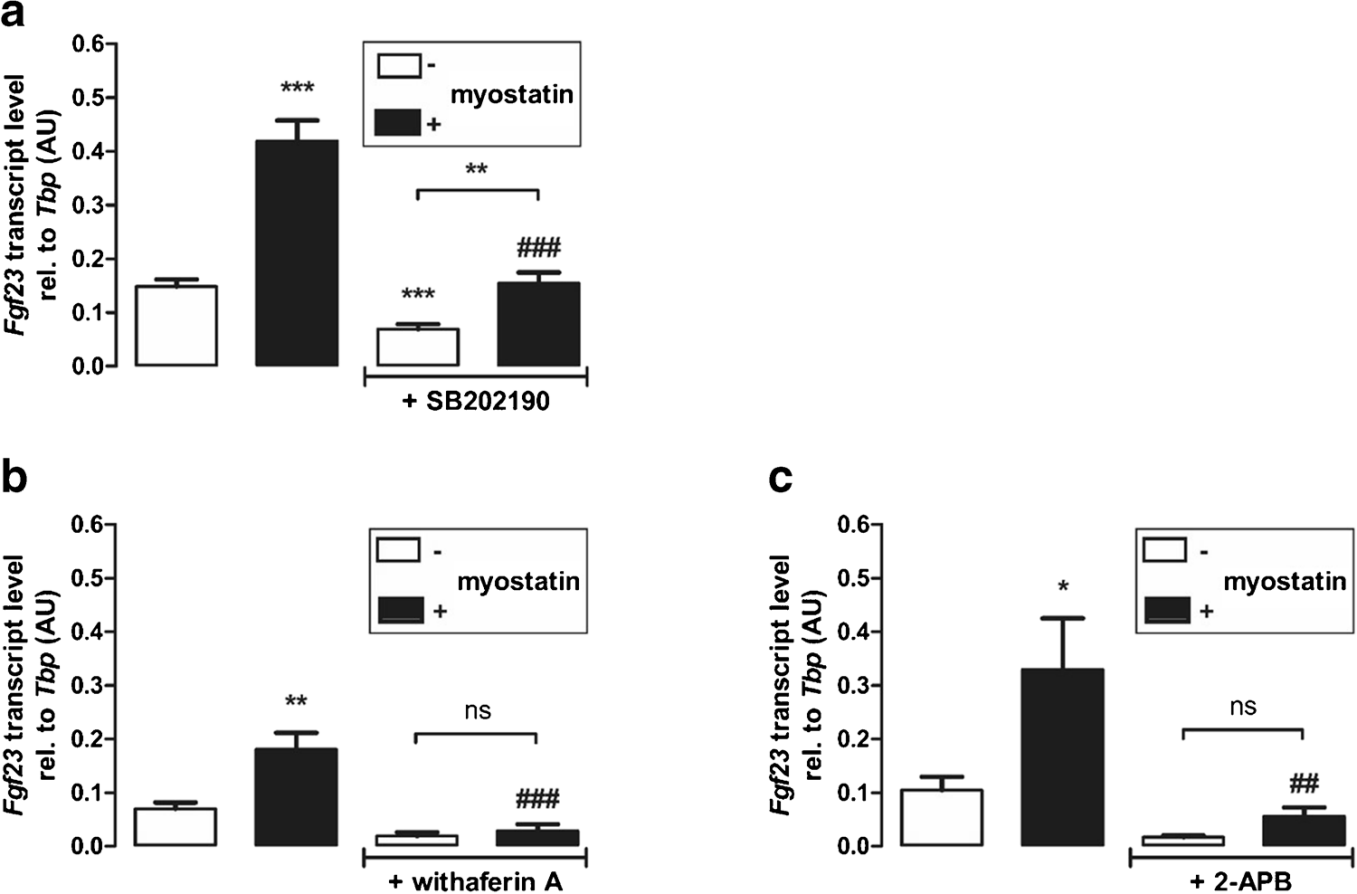
The myostatin effect on Fgf23 is at least in part dependent on p38MAPK and on NFκB-mediated store-operated Ca^2+^ entry (SOCE). Arithmetic means ± SEM of relative (rel.) *Fgf23* mRNA abundance in UMR106 cells treated without (white bars) or with (black bars) myostatin (100 ng/mL, 24 h) in the presence or absence of p38MAPK inhibitor SB202190 (**a** 10 µM, 24 h, n = 16) or NFκB inhibitor withaferin A (**b** 500 nM, 24 h, n = 6), or SOCE inhibitor 2-APB (**c** 150 µM, 24 h, n = 6). *p < 0.05, **p < 0.01, and ***p < 0.001 indicate significant difference from control. ##p < 0.01 and ###p < 0.001 indicate significant difference from the absence of **a** SB202190; **b** withaferin A, or **c** 2-APB (2nd vs 4th bar). AU, arbitrary units (**a** Welch’s ANOVA; **b**, **c** one-way ANOVA)

P38MAPK is a downstream target of myostatin [45] and also a potent regulator of FGF23 production [10]. To test whether p38MAPK contributes to the myostatin effect on *Fgf23* gene expression, UMR106 cells were incubated with or without 100 ng/mL myostatin in the presence or absence of 10 µM p38MAPK inhibitor SB202190 for 24 h. As demonstrated in Fig. 4a, SB202190 significantly blunted myostatin-mediated upregulation of *Fgf23* gene expression. Nevertheless, myostatin was capable of significantly enhancing *Fgf23* gene expression even in the presence of SB202190, pointing to the involvement of further effectors. Since pro-inflammatory transcription factor complex NFκB is a powerful regulator of FGF23 and myostatin induces NFκB activity [1], we performed further experiments to elucidate an involvement of NFκB. To this end, UMR106 cells were treated with and without 100 ng/mL myostatin in the presence or absence of NFκB inhibitor withaferin A (500 nM) for 24 h. As shown in Fig. 4b, myostatin did not significantly alter *Fgf23* gene expression in the presence of withaferin A.

NFκB enhances *Fgf23* gene expression by upregulating SOCE [47]. Hence, we sought to determine whether the myostatin effect is also dependent on SOCE. To this end, UMR106 cells were treated with or without myostatin in the presence or absence of SOCE inhibitor 2-APB (150 µM) for 24 h. No significant effect of myostatin on *Fgf23* gene expression was observed in UMR106 cells treated with 2-APB (Fig. 4c).

## Discussion

This study provides evidence that myostatin, a signaling molecule produced by skeletal muscle cells, is a potent regulator of the production of FGF23, a hormone produced by bone cells. According to our results, myostatin upregulated *Fgf23* gene expression and secretion of FGF23 protein in UMR106 osteoblast–like cells. Both, production of C-terminal and intact FGF23 were enhanced upon treatment with myostatin, suggesting that the myokine indeed regulates biologically active FGF23.

Myostatin was discovered as a myokine with mainly paracrine effects in skeletal muscle, i.e., the inhibition of skeletal muscle growth [16]. Our study adds to the growing concept of a muscle-bone cross-talk and further supports the notion of myostatin having paracrine and endocrine effects [25]. In this respect, it shares similarity with FGF23 which is also characterized by both paracrine and endocrine effects in different tissues and cells [28]. Other factors involved in this cross-talk are, among others, irisin, receptor activator of NF-κB ligand (RANKL), osteocalcin, sclerostin, or TGF-β [25, 29]. The regulation of bone-derived FGF23 through muscle-derived myostatin according to our study again underlines the mutual influence of bone and muscle. Interestingly, also IL-6 and TGF-β, other factors involved in bone-muscle cross-talk, are potent regulators of FGF23 [7, 13]. Conversely, bone-derived FGF23 also acts on skeleton muscle, inducing muscle atrophy [24, 29]

Our experiments also addressed the cellular mechanisms through which myostatin exerts its stimulatory effect on FGF23. The main membrane receptor for myostatin, ACVR2B [25, 38], was strongly expressed in UMR106 cells, whereas the expression level of ACVR2A was markedly lower. Conversely, expression of ACVR2B was low and that of ACVR2A was high in IDG-SW3 and MC3T3-E1 cells. Interestingly, myostatin failed to significantly affect *Fgf23* expression in these cells, a result in line with ACVR2B being the major mediator of the myostatin effect on FGF23. Moreover, our results suggest that TGF-βRI is involved, as TGF-βRI inhibitor SB431542 significantly attenuated the myostatin effect on *Fgf23*. In line with a decisive role of TGF-βRI signaling for the production of FGF23, an earlier study identified TGF-β as a major trigger of FGF23 formation [13].

**Fig. 5:**
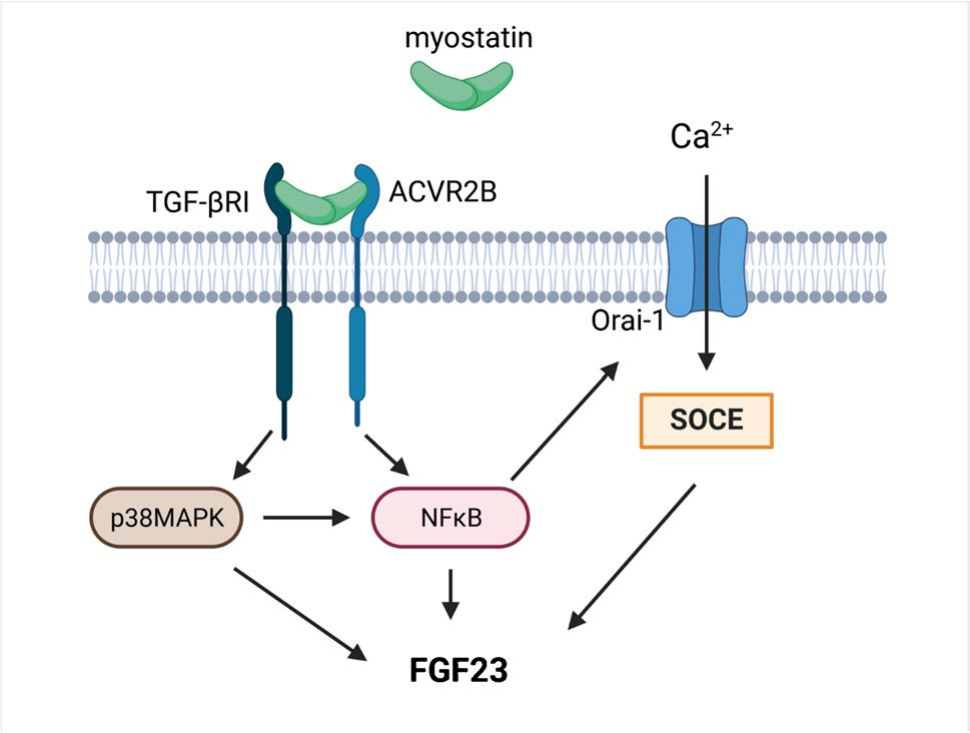
Schematic illustration of myostatin-induced FGF23 production in UMR106 cells. Myostatin binding to ACVR2B and following partnering with TGF-βRI activates p38MAPK and NFκB. NFκB induces SOCE, resulting in induction of *Fgf23* gene expression. Created with BioRender.com. Activin type 2 receptor B (ACVR2B); fibroblast growth factor 23 (FGF23); nuclear factor kappa-light-chain-enhancer of activated B-cells (NFκB); p38 mitogen–activated protein kinase (p38MAPK); store-operated Ca^2 +^ entry (SOCE); transforming growth factor-β type I receptor (TGF-βRI)

Downstream intracellular effectors of myostatin include p38MAPK [45]. Using p38MAPK inhibitor SB202190, we could demonstrate that also the myostatin effect on FGF23 is, at least in part, dependent on p38MAPK. This finding corroborates another study showing that p38MAPK signaling is a regulator of FGF23, in part through pro-inflammatory transcription factor complex NFκB [10]. Importantly, NFκB is a downstream target of myostatin [1] and itself is an important mediator of the stimulatory effect of inflammation and pro-inflammatory cytokines on FGF23 synthesis [22, 47]. NFκB is effective through inducing SOCE [47]. In line with this, both, NFκB and SOCE inhibition, significantly prevented myostatin from upregulating *Fgf23* gene expression. A summary of the putative signaling is presented in Fig. 5.

Myostatin not only prevents muscle hypertrophy but also an increase in bone mass as myostatin deficiency results in higher bone mass [9], bone mineral density and mineral content [34]. In line with this, myostatin inhibits osteoblast differentiation [37]. FGF23 induces a reduction of the plasma phosphate and 1,25(OH)_2_D_3_ concentration. Both effects are expected to favor a reduction in bone mineralization and mass [3]. Hence, the stimulatory effect of myostatin on FGF23 fits well into the concept of myostatin limiting skeletal muscle and bone mass.

In dermatomyositis, an inflammatory condition, the plasma myostatin concentration is elevated [23]. Importantly, the same study also found higher FGF23 levels in patients with dermatomyositis [23]. Moreover, CKD, another disease associated with elevated FGF23 levels [26], is characterized by a higher myostatin plasma concentration [46] and enhanced myostatin expression in muscle of mice [48]. These results are in line with our major finding, i.e., myostatin-dependent stimulation of FGF23 production.

Pharmacological manipulation of myostatin has already been tested as a therapeutic approach [25]: Myostatin inhibition may theoretically be beneficial in diseases with muscle weakness such as Duchenne muscular dystrophy or rheumatoid arthritis and was already tested [25]. According to our study, myostatin inhibition could result in lower FGF23, which may indeed be beneficial with regard to the reduced bone mass typical of both Duchenne muscular atrophy [4] and rheumatoid arthritis [30].

Data on the in vivo relevance of our in vitro results are sparse thus far: No gross differences in serum Ca^2+^ or phosphate were reported in Holstein Friesian calves and Belgian Blue calves [36]. Clearly, further in vivo studies exploring FGF23 and phosphate metabolism in myostatin-deficient mice or in Belgian Blue cattle are needed to confirm the significance of our findings.

Taken together, this study found a direct stimulatory effect of myostatin on *Fgf23* gene expression and protein production in UMR106 cells. Moreover, it uncovered that this effect is, at least in part, mediated by p38MAPK and NFκB. These findings may contribute to higher FGF23 levels in some diseases with enhanced myostatin production and may be relevant for future therapeutic approaches involving pharmacological manipulation of myostatin.

## Data Availability

Data and material will be shared.
